# Participatory co-design of pediatric rehabilitation technologies: a practice-based perspective

**DOI:** 10.3389/fmed.2026.1809380

**Published:** 2026-08-20

**Authors:** Arie Melamed Yekel, Naomi Gefen, Patrice L. Weiss

**Affiliations:** 1 ALYN Pediatric & Adolescent Rehabilitation Hospital, Jerusalem, Israel; 2 School of Occupational Therapy, Hebrew University, Jerusalem, Israel; 3 Department of Occupational Therapy, University of Haifa, Haifa, Israel

**Keywords:** co-design, digital health, human factors, implementation science, medical devices, participatory design, pediatric rehabilitation, regulatory science

## Abstract

Digital rehabilitation technologies are rapidly expanding across pediatric healthcare, yet many innovations fail to achieve sustained clinical adoption. In pediatric rehabilitation, this translation gap is amplified by heterogeneity in diagnoses and developmental stages, the need for family-centered care, and the practical constraints of clinical workflows. In this Perspective article, we argue that participatory co-design, an iterative process that actively involves clinicians, children, caregivers, and developers, incorporating stakeholder lived experience across the technology lifecycle, can reduce implementation failure and accelerate safe translation from concept to care. The purpose of this article is to present a structured, clinic-embedded participatory design framework spanning validated need identification, co-design, prototype testing, implementation support, and early-stage clinical validation. We illustrate this framework through two case examples from ALYN Pediatric & Adolescent Rehabilitation Hospital: 1. Headovations head support systems (Headaloft and the 360 multidirectional head support), and 2. the Pediatric Active-Passive Trainer (P-APT). Drawing on these experiences, we provide actionable recommendations for innovators, clinicians, and regulators regarding stakeholder engagement, usability and human factors evidence, documentation, and adoption metrics. We conclude that participatory co-design within pediatric rehabilitation hospitals represents a practical, regulatory-aligned pathway to foster technologies that are clinically meaningful, scalable, and sustainable in real-world care.

## 1 Introduction

Pediatric rehabilitation is increasingly influenced by advances in digital health, sensing technologies, and interactive therapeutic platforms (1). These innovations offer opportunities to enhance assessment, personalize intervention, and extend rehabilitation beyond the clinic. However, many pediatric rehabilitation technologies fail to achieve sustained clinical uptake (2, 3), reflecting a broader implementation gap in which interventions effective in controlled studies do not reliably translate into routine care (4, 5).

These barriers are amplified in pediatric rehabilitation by heterogeneity in diagnoses and developmental stages, and by the need to engage children, caregivers, and clinicians simultaneously.

In digital health, non-adoption and abandonment have been framed as sociotechnical challenges shaped by interactions between the technology, users, and service context (5). Implementation science frameworks such as the consolidated framework for implementation research (CFIR) provide structured approaches to identifying multilevel determinants of adoption and sustainability (6, 7). Child health technology research further emphasizes that pediatric innovation must address real-world barriers beyond efficacy, including stakeholder alignment and practical constraints that shape feasibility and adoption (8), and that children and young people have clear preferences and needs that influence sustained engagement and impact (9).

Importantly, usability and user experience are not solely intrinsic properties of a rehabilitation technology but are strongly influenced by the user's prior experience and the environment in which the technology is deployed, reinforcing the need to evaluate devices within realistic contexts of use. This has been demonstrated, for example, in wheelchair simulation research showing that both user experience level and environmental conditions can meaningfully shape usability outcomes and perceived user experience (10).

We argue that participatory co-design, i.e., iterative, clinic-embedded collaboration between end users and developers that explicitly incorporates end-user lived experience, offers a practical pathway to bridge this gap (11, 12). While participatory design is widely endorsed in principle, the literature provides limited granular descriptions of how it is operationalized across the full lifecycle of pediatric rehabilitation medical device development, including validated need identification, implementation and early validation. This perspective article addresses this need by presenting a structured framework and illustrating it through two practice-based case examples from ALYN Pediatric & Adolescent Rehabilitation Hospital.

## 2 Translation and implementation gap in pediatric rehabilitation technologies

Translation barriers in pediatric rehabilitation are amplified by heterogeneity in diagnoses and functional profiles, developmental change over time, and the need to engage both children and caregivers. In routine care, therapists must balance safety, engagement, and individualized goal-setting within constrained time and staffing resources; children may have limited attention, fatigue, sensory sensitivities, or communication challenges; and families must coordinate therapy alongside schooling and medical care. These realities can undermine adoption even when a technology appears effective (3, 6, 7).

Implementation failure commonly arises from (i) poor fit with clinical workflow (5), (ii) inadequate usability including insufficient accommodation of children's and families' lived experience in real-world contexts (13), (iii) insufficient training of caregivers and children; (iv) inadequate technical support, and (v) limited evidence on functional transfer to daily activities (14). From a regulatory science perspective, these factors also relate to human factors engineering, risk management, and documentation quality, issues that influence safety, acceptability, and scalability. These determinants motivate the five-stage framework below.

## 3 Participatory co-design is essential in pediatric rehabilitation

Participatory co-design extends beyond user feedback at the end of design. Instead, it embeds end users, including children, caregivers, and clinicians, throughout the innovation lifecycle (15). In pediatric rehabilitation, participatory approaches are particularly important because the end users are not a single individual. Rather, effective solutions must satisfy multiple stakeholder groups and incorporate their lived experience: children (engagement and accessibility), caregivers (burden and feasibility), clinicians (workflow and therapeutic relevance), and institutions (safety, governance, data protection, and sustainability) (16, 17). This requires an operational framework that can be embedded within clinical practice.

## 4 Structured framework for clinic-embedded participatory co-design

**Figure 1:**
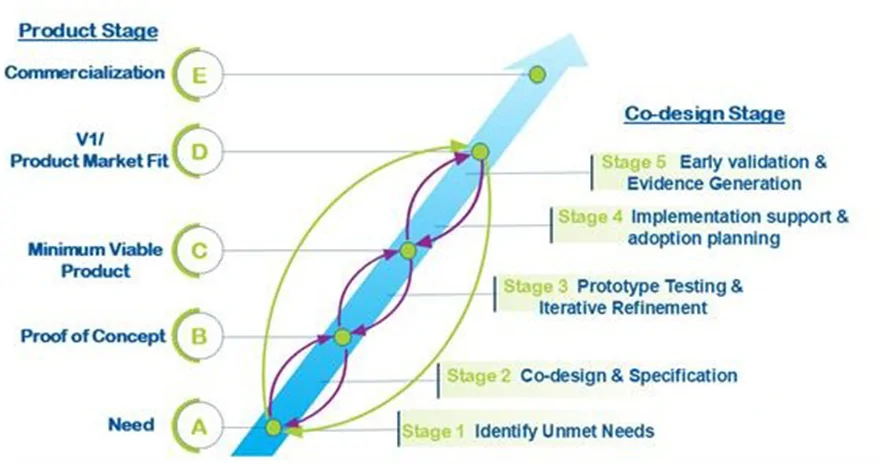
Five-stage, clinic-embedded framework for participatory co-design of pediatric rehabilitation technologies.

Based on our experience at ALYN Pediatric & Adolescent Rehabilitation Hospital, we propose a five-stage, clinic-embedded framework for participatory co-design of pediatric rehabilitation technologies. Figure 1 illustrates the staged co-design framework applied in this study, mapping the iterative clinical-development process onto conventional product stage maturity milestones (A**→**E). The model begins with identification of unmet needs (Stage 1) and progresses through co-design and specification (Stage 2), prototype testing with iterative refinement (Stage 3), and implementation/adoption planning (Stage 4), culminating in early validation and evidence generation (Stage 5). The figure emphasizes the bidirectional, feedback-driven nature of the process, highlighting how clinical input continuously informs successive technology iterations toward product-market readiness and commercialization.

This Co-design stage framework aligns with human-centered design standards and medical device usability engineering guidance and supports early identification of determinants of adoption such as compatibility, complexity, and perceived benefit. The stages are iterative rather than strictly linear and may require repeated cycles depending on technology maturity and clinical complexity.

### 4.1 Five-stage, clinic-embedded framework for participatory co-design

**Stage 1: Identify unmet needs**

- Define the clinical problem with end users (therapists, children, caregivers), informed by their lived experience. (18)
- Characterize the target population and context of use (clinic, school, home).
- Assess whether existing solutions are adequate; specify gaps and constraints.
- Translate needs into functional requirements and safety considerations.

**Stage 2: Co-design and specification** (15, 19)

- Conduct structured co-design sessions to refine use cases and user journeys.
- Develop measurable therapeutic objectives and define meaningful outcomes.
- Specify usability and accessibility requirements for pediatric users.
- Document design decisions and version changes to support traceability.

**Stage 3: Prototype testing and iterative refinement** (5)

- Implement rapid cycles of prototype testing in real clinical settings.
- Use mixed methods, including structured and semi-structured interviews, focus groups, direct observation during clinical use, standardized usability questionnaires (e.g., SUS, QUEST), and informal caregiver and child feedback captured during sessions.
- Identify barriers to workflow integration and child engagement early.
- Refine physical design, interface, and therapeutic content accordingly.

**Stage 4: Implementation support and adoption planning** (7)

- Train clinicians using short, role-specific formats (simulation preferred).
- Identify a clinical champion and define responsibilities.
- Ensure technical support pathways and device maintenance procedures.
- Define key performance indicators (KPIs) for usage and adoption (15).

**Stage 5: Early validation and evidence generation** (3, 14, 17)

- Select evaluation strategy proportional to risk and novelty (pilot vs. trial study).
- Measure usability, satisfaction, safety events, and adherence.
- Assess functional transfer and family-reported value.
- Use evidence to inform refinement, dissemination, and regulatory planning.

Across the five stages, stakeholder involvement should be tailored to the objectives of each stage rather than remaining constant. Clinicians are involved throughout all stages, while children and caregivers are primarily engaged during need identification, prototype testing, and early validation. Researchers and engineers support all stages, with intensity varying according to specific design and evaluation requirements. Typical participation includes 3–6 clinicians per stage, 5–15 children and caregivers during testing stages, and 2–4 researchers or developers supporting design, analysis, and documentation.

Importantly, we note that not all products or devices progress to Co-design Stage 5 or to Product Stage E, even when earlier stages are successfully completed. Attrition commonly reflects a combination of clinical, organizational, technical, and translational factors rather than a single point of failure. Early prototype testing may reveal limited functional benefit, insufficient transfer to daily activities, or poor fit with real-world clinical workflows, all of which are well-documented determinants of non-adoption and abandonment of health technologies (5). In other cases, progression is constrained by limited resources, including insufficient funding, lack of sustained industry engagement, or inadequate capacity for training and implementation support, factors highlighted in implementation science frameworks such as CFIR (7). Regulatory and evidence-related barriers may also halt advancement, particularly when human factors, safety, or usability evidence is insufficient to support early validation or scale-up (14). In pediatric rehabilitation, evolving clinical priorities and developmental heterogeneity further increase the likelihood that technologies are discontinued when alignment with stakeholder needs diminishes over time. Recognizing these realities reinforces the value of staged, participatory co-design as an evidence-informed decision-making process in which discontinuation at earlier stages may represent responsible translation rather than failure.

## 5 Case examples from ALYN pediatric & adolescent rehabilitation hospital

The following two examples illustrate how participatory co-design can be operationalized within a pediatric rehabilitation hospital environment. These cases represent different starting points: refinement of an emerging assistive technology (head support systems) and pediatric adaptation of an existing rehabilitation training device (active-passive trainer). The depth and formality of feedback collection varied across stages and case examples, reflecting pragmatic constraints of clinical practice; however, systematic documentation of stakeholder input was maintained throughout. The implementation of each product design and participatory co-design stage is detailed below and summarized in Table 1.

**Table 1:** Product design and participatory co-design stages for two example products.

Stage		Case 1	Case 2
Product design	Co-design	Headovations head support systems	Pediatric active–passive trainer
A. Need	1. Identify unmet needs	Initial concept pre-existed; ALYN clinicians refined unmet needs during use (e.g., sub-occipital support, midline control, multi-planar stability), with needs revisited iteratively.	Clinicians explicitly defined pediatric unmet needs, including safety, anthropometrics, accessibility, and workflow integration.
	2. Co-design & specification	Clinicians, caregivers, and designers translated functional needs into design and engineering specifications.	Structured co-design sessions specified pediatric goals, usability, and safety requirements.
B. Proof of concept	3. Prototype testing & refinement	Prototypes tested in real clinical use; informal and structured feedback from children, caregivers, and clinicians guided iterative refinement.	Two prototypes evaluated in clinical sessions using SUS, QUEST, observation, and interviews to inform refinement.
C. Minimum viable product D. Product market fit	4. Implemen-tation & adoption	Integrated into ALYN lending program; clinical guidelines supported safe and consistent adoption.	Pilot implementation informed workflow integration and manufacturer adoption planning.
E. Commer-cialization	5. Early validation	Year-long evaluation demonstrated high usability and functional benefit for families.	Pilot results demonstrated moderate-to-good usability and directly informed next-generation device development.

### 5.1 Headovations head support systems (Headaloft and 360) (20)

HEADALOFT™ and the 360 multidirectional head support systems (https://www.gal-kal.co.il/about/) provide solutions for comfortable and stable head support for children when sitting in a wheelchair, car seat, stroller or any other device equipped with a headrest. They greatly improve feelings of discomfort in the neck, allow for easier breathing, facilitate feeding and swallowing food and drink as well as better eye-focusing communication. The development of these devices illustrate a sustained participatory co-design process between ALYN clinicians, the Headovations founder/CEO, and an industrial product designer. Although the original Headaloft concept was initiated externally, the refinement process was guided by real-world pediatric clinical needs identified by occupational therapists at ALYN. Clinicians emphasized the need for sub-occipital support to prevent hyperextension and improve midline orientation—an element not sufficiently addressed in early prototypes. This clinical requirement became a defining feature of the final design. This study was approved by the Helsinki Committee of ALYN Pediatric and Adolescent Rehabilitation Hospital (#053-21). Written informed consent to participate in this study was provided by the participants' legal guardian/next of kin. Where applicable, assent was obtained from the children in accordance with institutional guidelines.

HeadAloft prototypes were tested with children at ALYN while they used their own mobility devices with headrests, such as wheelchairs, strollers, and standers. HeadAloft is designed as an add-on accessory that attaches to these devices. Feedback from children, parents, and therapists (including families' lived experience of daily mobility challenges) was collected through informal interviews during device trials, structured clinician discussions, and parent-reported experiences gathered as part of the lending program. This informed iterative improvements in comfort, positioning, and functional impact. For example, this headrest incorporates an anterior forehead strap that can be repositioned superiorly and displaced off the forehead without full detachment, minimizing the risk of misplacement or loss. This functionality is enabled by rotational joints at the strap attachment points, allowing controlled movement of the strap ends. In parallel, the 360 head support system was developed to address the needs of children with fluctuating tone, involuntary movements, dystonia, and asymmetric posture. ALYN clinicians articulated functional requirements for multi-planar adjustability and stability, which were translated into engineering specifications.

The 360 system and HeadAloft were integrated into ALYN's lending program https://www.alyn.org/project-for-loaning-head-rests and evaluated through a year-long data collection effort (February 2023–February 2024). Families reported high usability and functional benefit, supporting the clinical value of multidirectional head support for complex pediatric wheelchair users. In addition to device refinement, the collaboration supported development of clinical guidelines for head support assessment and selection, strengthening safe implementation.

In this case example, the participatory process did not begin at Stage 1 (unmet need identification), as the initial product concept was initiated previously. Instead, the framework was applied from Stage 2 onward, with clinicians, children, and caregivers contributing primarily to co-design, prototype testing, implementation refinement, and early validation (Stages 2–5). Elements of Stage 1 were subsequently revisited as clinical experience revealed additional unmet needs.

### 5.2 Pediatric active-passive trainer (P-APT)

An active–passive trainer is a rehabilitation device that enables people with motor impairments to perform repetitive limb movements either actively with voluntary effort or passively with motor-assisted motion to support motor recovery and functional improvement (21, 22). Over an eight-month period, ALYN clinicians and engineers worked with the Pediatric Active-Passive Trainer (P-APT) https://www.tzora.com/2apt/ to use participatory co-design to support pediatric adaptation of an existing rehabilitation technology. We conducted seven structured working sessions to define pediatric therapeutic goals, operational constraints, and safety specifications. Key considerations included anthropometric variability across ages, accessibility for children with limited hand function, and therapist workflow integration. This study was approved by the Helsinki Committee of ALYN Pediatric and Adolescent Rehabilitation Hospital (#066-22). Written informed consent to participate in this study was provided by the participants' legal guardian/next of kin. Where applicable, assent was obtained from the children in accordance with institutional guidelines.

These co-design sessions informed modifications to the adult device, including scaled dimensions, improved ergonomics, and a simplified interface with basic game-based activities intended to enhance motivation. Two prototypes were delivered and evaluated in clinical use. Pilot implementation included children using the device either during a single session or over a two-week period as part of their rehabilitation program.

User feedback was collected using standardized questionnaires, complemented by qualitative feedback from therapists, parents, and children obtained through interviews and observational notes during clinical sessions. Usability and satisfaction were assessed using standardized measures (System Usability Scale (SUS) and Quebec User Evaluation of Satisfaction with Assistive Technology (QUEST)) combined with qualitative feedback capturing user lived experience from therapists, parents, and children. Findings indicated moderate-to-good usability, with therapists reporting the highest satisfaction. Parents emphasized the need for greater adjustability and stability, and children highlighted that limited game variety reduced sustained engagement. These results provided clear priorities for continued refinement before broader dissemination.

Following completion of the pilot study and subsequent review of findings, the company used the reported priorities, particularly those related to adjustability, stability, and usability within routine clinical workflows, to inform the next iteration of the technology. Most notably, they developed an integrated configuration termed the “Double APT,” which combines an upper-limb and lower-limb APT into a single system. The pediatric-adapted APT co-designed through the ALYN-led process served as the foundational module for both upper- and lower-limb components of this combined platform, demonstrating direct translation of study-driven refinements into the manufacturer's subsequent product architecture.

In parallel, post-study market uptake and user feedback indicated that many purchasers, including adult users, prefer the smaller, more compact APT form factor developed through the pediatric adaptation, citing improved portability and a more streamlined design. In contrast, the game-based interface elements, initially introduced to enhance motivation and engagement, have not emerged as a primary driver of adoption and have not been further expanded. This observation is consistent with real-world use patterns: across both adult and pediatric versions, many users rely predominantly on passive training modes, in which the game features are not operational. Collectively, these findings suggest that the device's primary value derives less from software-based engagement components and more from compact, robust hardware that supports passive and therapist-guided therapeutic application, thereby clarifying priorities for future development and dissemination.

## 6 Discussion

From a regulatory science perspective, participatory co-design can be conceptualized as an evidence generation strategy that strengthens traceability, usability engineering, and integrates stakeholder lived experience as part of the context-of-use evidence across the device lifecycle (23). This perspective is consistent with recent calls in disability and rehabilitation research to adopt faster, staged, and pragmatic approaches for generating evidence for technology-based interventions, particularly in rapidly evolving innovation contexts (14). The five-stage framework presented here complements existing participatory design literature by providing a clinic-embedded operational model that integrates implementation planning and early-stage validation (3, 24). Importantly, participatory processes should be documented as part of design history and risk management files, supporting regulatory-aligned translation of pediatric rehabilitation technologies (5, 9).

Early-stage needs assessment is particularly critical in pediatric assistive technology, where device success depends on alignment with child-specific anthropometrics, functional abilities, and caregiver and clinician expectations. Explorative work to define user needs for pediatric upper-limb assistive devices underscores the importance of systematically capturing these requirements prior to design finalization (25).

### 6.1 Lessons learned and actionable recommendations

Across both cases, several practical lessons emerged that support more sustainable translation of pediatric rehabilitation technologies:

- Begin with validated unmet needs and define measurable clinical objectives before building solutions (stage 1).
- Design for workflow integration: setup time, documentation burden, and therapist cognitive load are adoption determinants (stages 2 and 3).
- Engage end users (children, caregivers, and clinicians) throughout all five stages, with a central role in usability and acceptability testing during iterative prototype refinement (stages 1–5).
- Document iteration decisions and version changes to support transparency, traceability and regulatory alignment (stages 2 and 3).
- Plan implementation early: training, technical support, and maintenance pathways must be defined before scale-up (stage 4).
- Track adoption KPIs (e.g., frequency of use, completion rates, dropout reasons) alongside clinical outcomes (stage 4).
- Match evidence generation to risk: low-risk modifications may require pragmatic pilots; higher-risk innovations may require controlled trials (stage 5).

### 6.2 Toward rigorous evaluation of participatory design

While participatory co-design is increasingly recognized as necessary for aligning pediatric rehabilitation technologies with real-world needs and contexts (8, 9), there remains a critical need to rigorously evaluate the effectiveness of participatory methods themselves rather than assuming their value (14). Future research should adopt mixed-methods and implementation science approaches that systematically compare outcomes from projects using participatory design with those that do not, using standardized metrics such as user engagement, usability outcomes, adoption rates, and long-term sustainability (26). For example, social science frameworks like realist evaluation can help elucidate *what works, for whom, and under what conditions* by linking participatory processes to measurable implementation outcomes (27). Additionally, randomized or quasi-experimental designs embedded within development processes can assess whether iterative stakeholder involvement leads to superior adoption, reduced abandonment, or better clinical outcomes compared to standard development pathways (5–7). To facilitate this, future work should define clear participatory design process indicators (such as diversity of stakeholder input, frequency of iteration cycles, and documentation quality) and relate them to downstream evidence needs important to regulators, clinicians, and patient representatives. Establishing such an evidence base will strengthen the case for participatory co-design as a strategy not only theoretically preferable but demonstrably effective in accelerating safe, scalable, and impactful pediatric rehabilitation technologies.

## 7 Conclusions

Our five-stage framework is consistent with broader pediatric assistive technology development literature emphasizing that user requirements should be explicitly defined through structured stakeholder input prior to technical refinement, including exploratory surveys and needs-definition studies in pediatric exoskeleton development (25). Pediatric rehabilitation technologies are unlikely to achieve sustained impact without explicit attention to usability, workflow integration, and implementation support. Our five-stage, clinic-embedded participatory co-design framework and illustrated its feasibility through two case examples of technologies for pediatric rehabilitation. This approach offers a practical pathway for generating regulatory-aligned evidence that supports safe translation and real-world adoption.

## Data Availability

The raw data supporting the conclusions of this article will be made available by the authors, without undue reservation.
